# Knockout of the peroxiredoxin 5 homologue *PFAOP* does not affect the artemisinin susceptibility of *Plasmodium falciparum*

**DOI:** 10.1038/s41598-017-04277-5

**Published:** 2017-06-30

**Authors:** Carine F. Djuika, Verena Staudacher, Cecilia P. Sanchez, Michael Lanzer, Marcel Deponte

**Affiliations:** Department of Parasitology, Ruprecht-Karls University, Im Neuenheimer Feld 324, D-69120 Heidelberg, Germany

## Abstract

Artemisinins are the current mainstay of malaria chemotherapy. Their exact mode of action is an ongoing matter of debate, and several factors have recently been reported to affect an early stage of artemisinin resistance of the most important human malaria parasite *Plasmodium falciparum*. Here, we identified a locus on chromosome 7 that affects the artemisinin susceptibility of *P. falciparum* in a quantitative trait locus analysis of a genetic cross between strains 7G8 and GB4. This locus includes the peroxiredoxin gene *PFAOP*. However, steady-state kinetic data with recombinant *Pf*AOP do not support a direct interaction between this peroxidase and the endoperoxide artemisinin. Furthermore, neither the overexpression nor the deletion of the encoding gene affected the IC_50_ values for artemisinin or the oxidants diamide and *tert*-butyl hydroperoxide. Thus, *Pf*AOP is dispensable for blood stage parasite survival, and the correlation between the artemisinin susceptibility and chromosome 7 is probably based on another gene within the identified locus.

## Introduction

**Figure 1:**
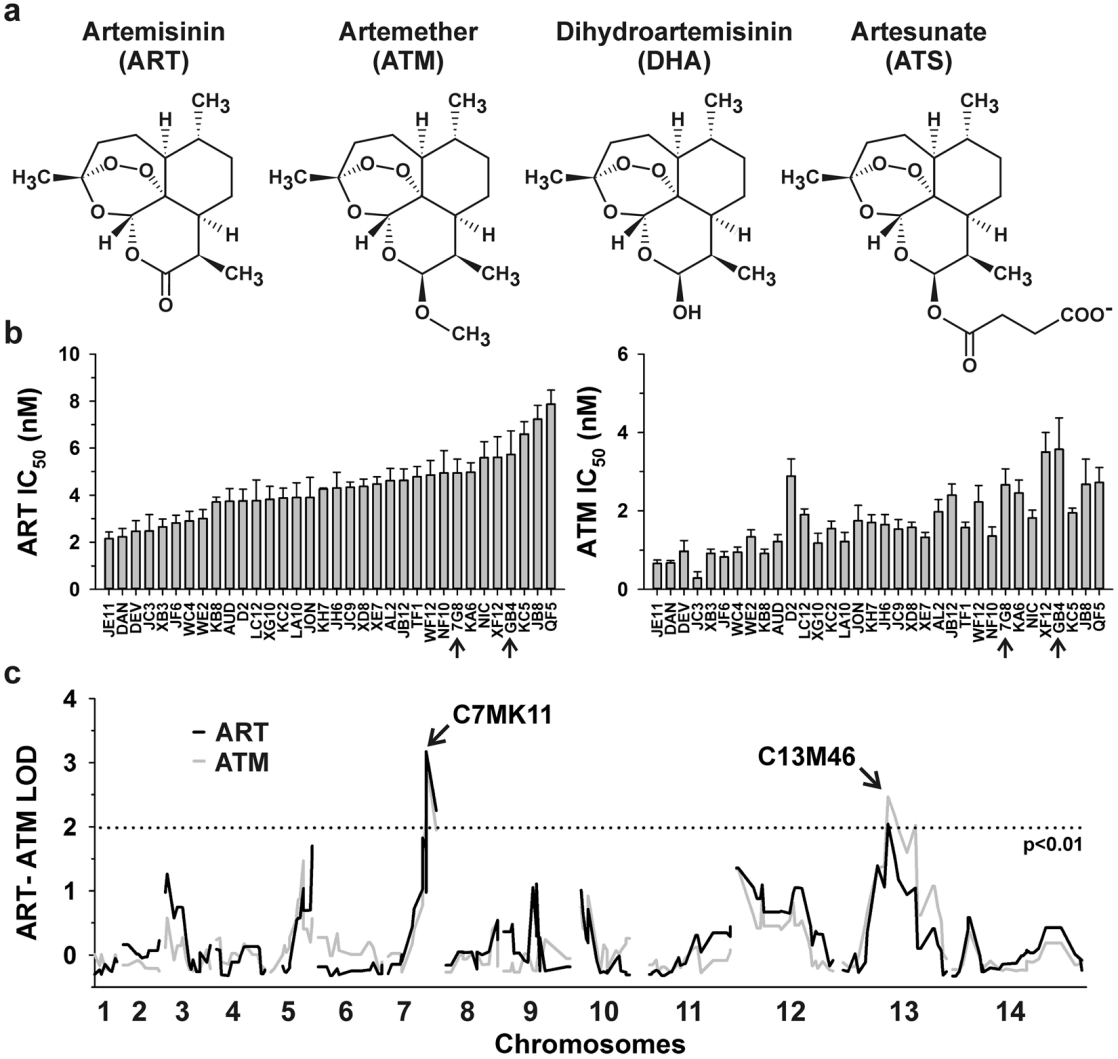
Chemical structures of artemisinin derivatives and QTL analysis. (**a**) Chemical structures of compounds indicated. (**b**) *In vitro* ART and ATM IC_50_ values of 32 F1 progeny from the 7G8 × GB4 cross and the two parental clones (indicated by arrows). Means ± SEM of at least five independent determinations. (**c**) QTL analysis. The logarithm of odds (LOD) scores are shown for ART (black lines) and ATM (grey lines) responses as a function of genomic location. Loci associated with decreased ART and ATM susceptibility are indicated. The dotted line represents the confidence line with p < 0.01.

Artemisinin from the herb *Artemisia annua* is a traditional, highly efficient antimalarial drug^1, 2^. Artemisinin-based combination therapies have been recommended by the World Health Organization for the treatment of *Plasmodium falciparum* malaria since 2001 and have become a key factor of global malaria control programs. For example, artemisinin and its derivatives (Fig. 1a) are estimated to reduce mortality in young children with uncomplicated malaria by more than 95%^3, 4^. However, early stage artemisinin resistance has emerged in form of a delayed parasite clearance phenotype^5^. The altered artemisinin susceptibility has been associated with discrete mutational changes within a gene that encodes the *P. falciparum* kelch protein K13^6–8^. Mutations in the K13 gene alone cannot account for all resistance cases, suggesting that delayed parasite clearance is the result of convergent evolutionary events^8, 9^. Additional factors that might affect the artemisinin susceptibility of *P. falciparum* or facilitate K13 mutations were identified by whole genome sequencing of blood samples as well as reverse genetics and chemogenomic profiling *in vitro* ^8, 10, 11^. A quantitative trait locus (QTL) analysis also revealed an effect of the parasite’s multidrug resistance transporter PfMDR1 and of two additional loci on chromosomes 12 and 13 on artemisinin susceptibility^12^.

The exact mode(s) of action of artemisinin as well as the mechanisms that contribute to artemisinin resistance are still a matter of debate and might be even interconnected. Numerous potential targets – including lipids and proteins such as the phosphatidylinositol-3-kinase PfPI3K – have been recently identified and localise to a variety of subcellular compartments^13–18^. Transcriptome analyses suggest that artemisinin resistance is linked to the lipid and protein metabolism of *P. falciparum* resulting in an altered cell cycle progression in accordance with experiments in cell culture^11, 19–22^. The activity of artemisinin depends on its endoperoxide moiety (Fig. 1a), which was suggested to be reduced and activated by heme-bound Fe^2+^ within the digestive vacuole, where the proteolytic degradation of hemoglobin takes place^16, 18, 23, 24^. Furthermore, artemisinin was hypothesized to promote the formation of reactive oxygen species resulting in so-called oxidative stress^18, 24–27^, although such an unspecific mode of action has also been questioned^13, 23^. In this regard it is interesting to note that the toggling of redox switches in molecular sensors and transducers is a specific process^28^, and that K13 shares a high similarity with Keap1, which is the primary redox sensor for Nrf2-dependent redox signalling in mammals^7, 29^. Whether K13 is involved in similar redox signaling cascades in *P. falciparum* is unknown.

Other crucial sensors in redox signalling belong to the ubiquitous protein family of peroxiredoxins (Prx). These hydroperoxidases catalyse the reduction of H_2_O_2_ and other hydroperoxides yielding water and the corresponding alcohol as products. Some Prx also exert functions as chaperones^30–32^. The *P. falciparum* genome encodes five different Prx-isoforms^33–36^. Furthermore, the parasite was shown to import functional human Prx-2 from erythrocytes^37^. According to current theories, *Plasmodium* Prx detoxify hydroperoxides in different subcellular compartments^34, 35, 38–41^. However, experimental data supporting the relevance of the *Plasmodium* Prx-isoforms for parasite survival is rather limited. While the cytosolic isoforms TPx-1 and 1-Cys-Prx were shown to be dispensable for the development of *P. falciparum* and *P. berghei* blood stage parasites^42–44^, attempts to delete the gene that encodes the nuclear isoform nPrx were unsuccessful and point towards an essential function^35^. The relevance of the other two *Plasmodium* Prx-isoforms for parasite survival remains unclear. One of these isoforms is the so-called antioxidant protein *Pf*AOP which belongs to the Prx5 subfamily and is found in the plastid (apicoplast) and cytosol of *P. falciparum* ^41^. The dual localization of *Pf*AOP presumably arose from a gene fusion event following a horizontal prokaryote-to-eukaryote gene transfer^41^. Although studies on recombinant *Pf*AOP confirmed its peroxidase activity *in vitro* ^45–47^, its physiological relevance for hydroperoxide removal or redox homeostasis has not been addressed before.

Here we identified a locus on chromosome 7 that affects the artemisinin susceptibility in the genetic cross between the *P. falciparum* clones 7G8 and GB4. One of the 49 genes within the locus encodes *Pf*AOP. Taking into account the established anti-oxidative activity of *Pf*AOP and the hypothesized pro-oxidative mode of action of artemisinins, we investigated a potential contribution of *Pf*AOP to artemisinin susceptibility and parasite survival.

## Results

### QTL mapping reveals *PFAOP* as a potential artemisinin susceptibility factor

In a previous study we have interrogated the contribution of the genetic background on artemisinin susceptibility by investigating artemisinin responses in the progeny of a genetic cross between the *P. falciparum* clones HB3 (Latin America) and Dd2 (Southeast Asia). We found that loci on chromosomes 5 (including Pfmdr1), 12 and 13 contribute to altered responsiveness^12^. Here we repeated the study, but this time using the genetic cross between strains 7G8 (Latin America) and GB4 (Africa)^48^. Responses to artemisinin and artemether were determined, using a standard cell proliferation assay where parasites were exposed to the drugs for 72 h^49^. The IC_50_ values of strains 7G8 and GB4 were for artemisinin 5.0 ± 0.6 nM and 5.7 ± 1.0 nM and for artemether 2.7 ± 0.4 nM and 3.6 ± 0.8 nM, respectively. Although the parental clones had comparable IC_50_ values for artemisinin and artemether, the responsiveness of the 32 progeny investigated covered a range from 2.1 ± 0.3 nM to 7.9 ± 0.6 nM in the case of artemisinin and from 0.3 ± 0.1 nM to 3.6 ± 0.8 nM in the case of artemether (Fig. 1b).

To identify determinants of altered artemisinin and artemether responsiveness, we conducted a QTL analysis by correlating the IC_50_ values with the genetic maps of the progeny and the parental clones^48^. One major QTL with a LOD score of 3.1 (p < 0.001) was identified for both drugs, mapping to the genetic marker C7MK11 on chromosome 7. According to the genome of the reference strain 3D7 this locus encodes 49 genes including *PFAOP* (PF3D7_0729200) (Table S1). The locus is distinct from the artemisinin susceptibility loci identified in the HB3 × Dd2 cross and it is also distinct from the pfcrt locus on chromosome 7 that is associated with chloroquine resistance and altered susceptibility to quinine and quinine-like antimalarial drugs^50–52^. We found no link with the K13 gene on chromosome 13^7^ or the PfPI3K gene on chromosome 5^15^. This finding was anticipated since the two parental clones carry identical wild type loci of these two genes.

### Artemisinins are neither substrates nor inhibitors of *Pf*AOP

**Figure 2:**
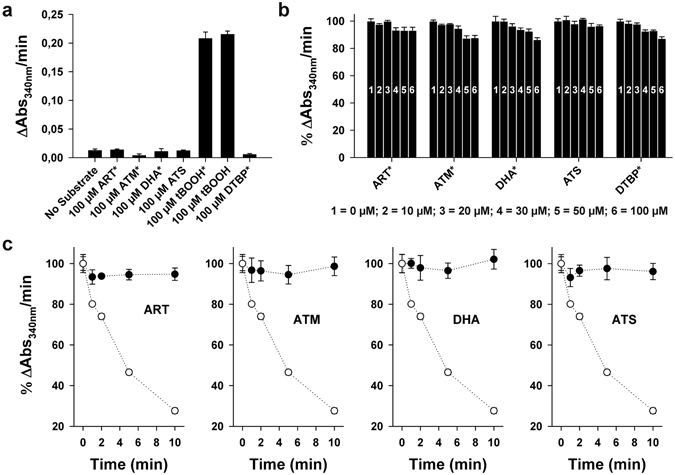
Peroxidase activity of recombinant *Pf*AOP in the presence of artemisinins. (**a**) Coupled enzymatic assays with 2.5 µM *Pf*AOP and artemisinins as potential substrates. Activities with 100 µM tBOOH or DTBP served as controls. (**b**) Reversible inhibition assays in the presence of variable concentrations of artemisinins or DTBP. Activities were determined with 2.5 µM *Pf*AOP and 50 µM tBOOH and normalized to the activities of controls without endoperoxide. (**c**) Time-dependent irreversible inhibition assays. *Pf*AOP (50 µM) was preincubated with 100 µM artemisinins for up to 10 min on ice before the enzyme was diluted 1:20 in a standard coupled enzymatic assay containing 100 µM tBOOH. Activities were normalized to controls that were preincubated without peroxide. A time-dependent inactivation of *Pf*AOP by tBOOH served as a positive control (open circles) and was compared to a potential inactivation by artemisinins (closed circles). Assays containing DMSO are labelled with an asterisk. All data are the mean ± standard deviation of at least two independent duplicate assays.

In order to test a potential direct interaction between artemisinins and *Pf*AOP, we first analysed whether recombinant *Pf*AOP converts endoperoxides in an established coupled enzymatic assay (Fig. 2a). The detected consumption of NADPH in the presence of artemisinins or di-*tert*-butyl peroxide (DTBP) was comparable to the background activity of negative controls without peroxide substrate, whereas the conversion of *tert*-butyl hydroperoxide (tBOOH) served as a positive control as described previously^45, 47^. Thus, *Pf*AOP does not convert endoperoxides and its enzymatic activity is restricted to hydroperoxide substrates.

Next, we analysed whether *Pf*AOP is inhibited by artemisinins. A reversible competitive inhibition was tested for the conversion of tBOOH in the presence of variable concentrations of artemisinins (Fig. 2b). Furthermore, a time-dependent irreversible inhibition was addressed by preincubating recombinant *Pf*AOP with artemisinins, DTBP or tBOOH before transferring the enzyme to standard assays with tBOOH (Fig. 2c). No inhibitory effects were detected except for the positive control with tBOOH, which is not only a substrate but also an irreversible inhibitor that ‘overoxidizes’ *Pf*AOP^45, 47^. In summary, artemisinins are neither reversible nor irreversible inhibitors of recombinant *Pf*AOP and enzymatic assays do not support a direct effect of *Pf*AOP on artemisinins and *vice versa*. However, our enzymatic assays neither exclude a potential interaction between *Pf*AOP and Fe^2+^-activated artemisinin nor indirect effects of *Pf*AOP on the artemisinin susceptibility of *P. falciparum*. In order to address these aspects, we genetically manipulated the reference strain 3D7 and determined the artemisinin susceptibility of overexpressing and knockout strains.

### Overexpression of *PfAOP* does not alter the artemisinin susceptibility

**Figure 3:**
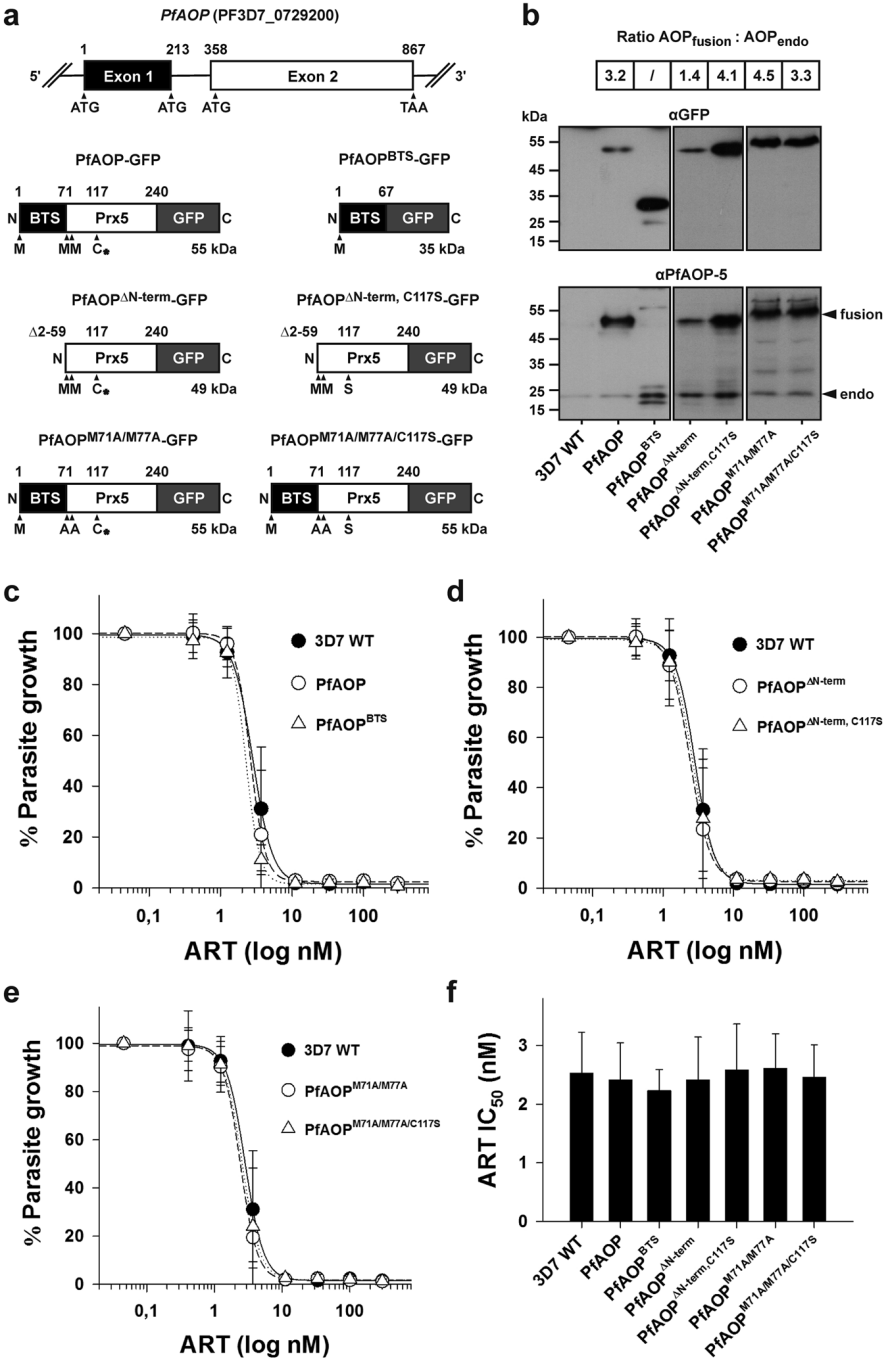
IC_50_ values for artemisinin of parasites that overexpress *PfAOP*. (**a**) The modular gene architecture of *PfAOP* is shown on top. Exon 1 encodes the bipartite topogenic signal (BTS) and exon 2 the Prx5 domain. Constructs encoding cytosolic truncated as well as apicoplast-targeted full length GFP-fusion proteins of *Pf*AOP are depicted below and were overexpressed in strain 3D7 as described previously^41^. Residue Cys117 at the active site of *Pf*AOP was shown to be essential for the hydroperoxidase activity^45^ and is labelled with an asterisk. The residue was mutated to serine in controls. (**b**) Western blot analyses confirming the overexpression of the GFP-fusion constructs listed in panel (a). Detection of GFP in the upper blot revealed no significant degradation of the fusion proteins. Hence, detection of *Pf*AOP at approximately 22 kDa reflects only endogenous *Pf*AOP (endo) whereas the upper bands in the same blot reflect the GFP-fusion constructs (fusion). Representative images were obtained by standard western blotting. The estimated ratio between both protein species is summarized on top and is based on semi-quantitative western blotting using a C-DiGit Blot Scanner in parallel experiments. (**c**–**e**) Artemisinin dose-response curves of strain 3D7 blood stage cultures that express the indicated GFP-fusion constructs from panel a. All data are the mean ± standard deviation of at least three independent triplicate measurements. (**f**) IC_50_ values obtained from panels c-e. None of the differences between the IC_50_ values was found to be significant (p > 0.05) based on statistical analyses in SigmaPlot 12.5 using the one way ANOVA method.

Artemisinins have been suggested to promote the formation of reactive oxygen species including H_2_O_2_^18, 24–27^. The removal of H_2_O_2_ or other hydroperoxides by *Pf*AOP might, therefore, affect the artemisinin susceptibility of *P. falciparum*. *Pf*AOP has a bipartite topogenic signal (BTS), which is required for correct apicoplast targeting of the dual localized protein^41^. In order to test a potential role of the cytosolic and/or apicoplast form of *Pf*AOP, we sequenced exon 1 (which encodes the BTS) of the parental strains and selected progenies. No single nucleotide polymorphisms or other mutations were found. Next, we overexpressed cytosolic as well as apicoplast-targeted truncated or full length GFP-fusion constructs of *Pf*AOP in 3D7 wild type cells as described previously^41^. In addition, we generated and analysed enzymatically inactive serine mutants^45^ of these constructs as controls (Fig. 3a). All GFP-fusion constructs were successfully expressed in *P. falciparum* resulting, on average, in an approximately 2- to 6-fold increase of the total cellular *Pf*AOP content (Fig. 3b). None of the overexpressed constructs led to an increase of the IC_50_ value for artemisinin or had a dominant-negative effect (Fig. 3c–f). In summary, irrespective of the protein localization or the presence or absence of the catalytic cysteine residue, overexpression of *PFAOP* does not alter the artemisinin susceptibility of *P. falciparum*.

### *Pf*AOP is not essential and does not protect against artemisinin or external oxidants

**Figure 4:**
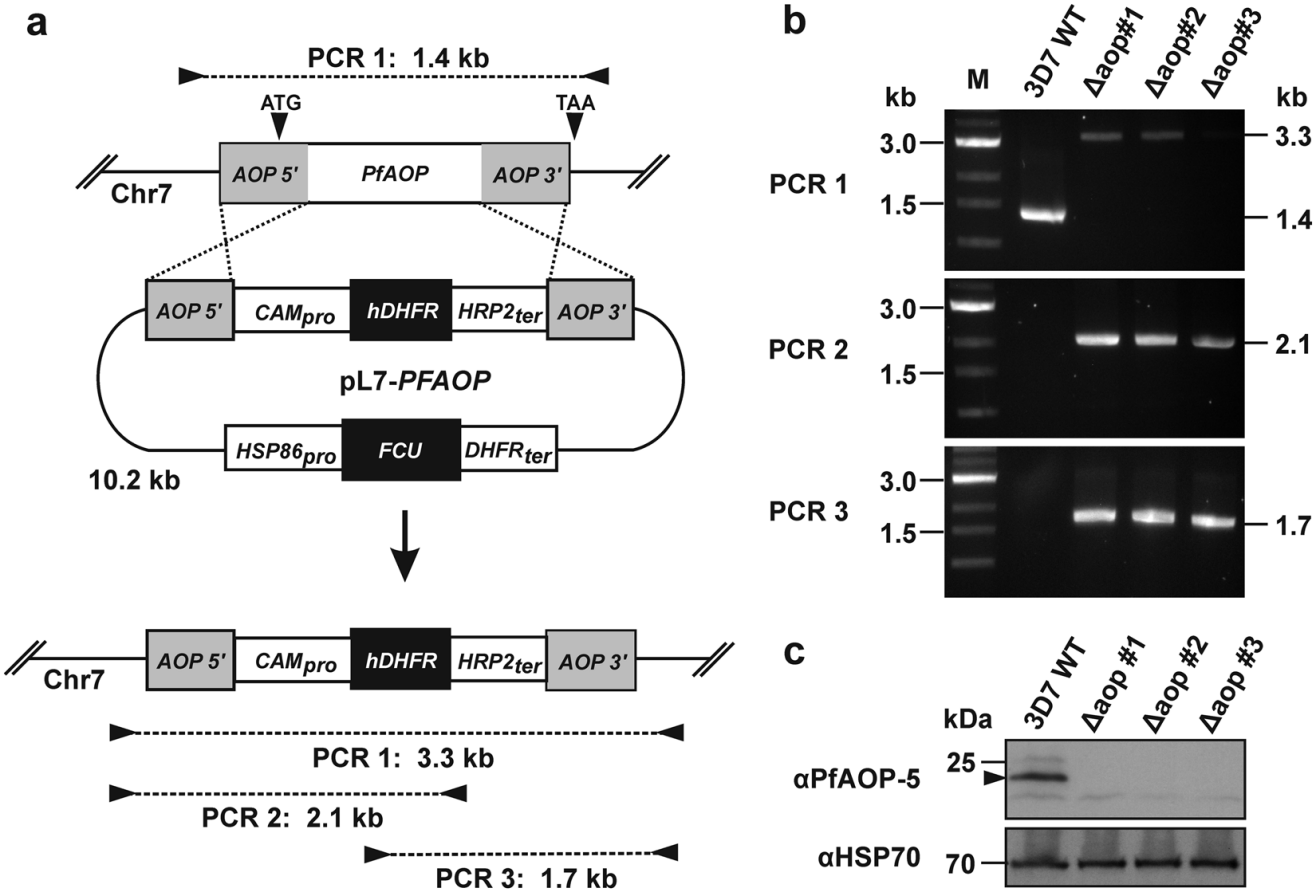
Generation and validation of Δ*pfaop* knockout parasites. (**a**) Schematic summary of the knockout strategy by double crossover using the plasmid pL7-*PFAOP*. Expected product sizes from analytical PCR reactions 1–3 are highlighted. The two external primers should anneal to chromosome 7, whereas the two internal primers should anneal to the gene that encodes the selection marker human dihydrofolate reductase (hDHFR). (**b**) Following the genetic manipulation of the wild type strain 3D7 (3D7 WT) and isolation of three clonal cell lines (Δaop#1-3), products from PCR reactions 1-3 were analysed by agarose gel electrophoresis. Marker (M) and expected product sizes are indicated on the left and right side, respectively. (**c**) Western blot analysis of parasite cell lines Δaop#1-3. Protein extracts from 10^7^ parasites were loaded per lane. Hsp70 was decorated as a control.

**Figure 5:**
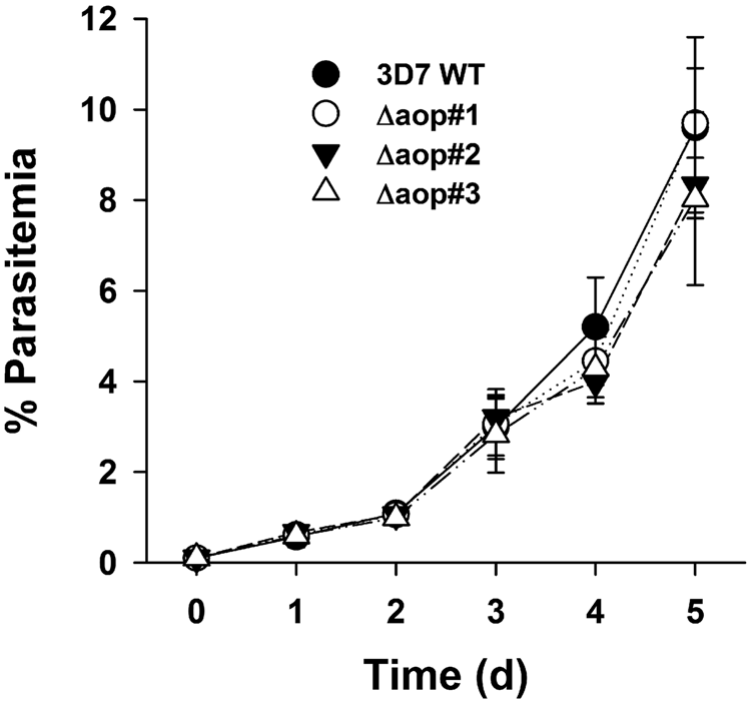
Growth curve analysis. The parasitemia in standard blood stage cultures of strains Δaop#1-3 from Fig. 4 was determined by counting parasites from Giesma-stained blood smears. Wild type strain 3D7 served as a control. All data are the mean ± standard deviation of triplicate measurements for each strain. None of the differences between the parasitemias was found to be significant (p > 0.05) based on statistical analyses in SigmaPlot 12.5 using the one way ANOVA method.

**Figure 6:**
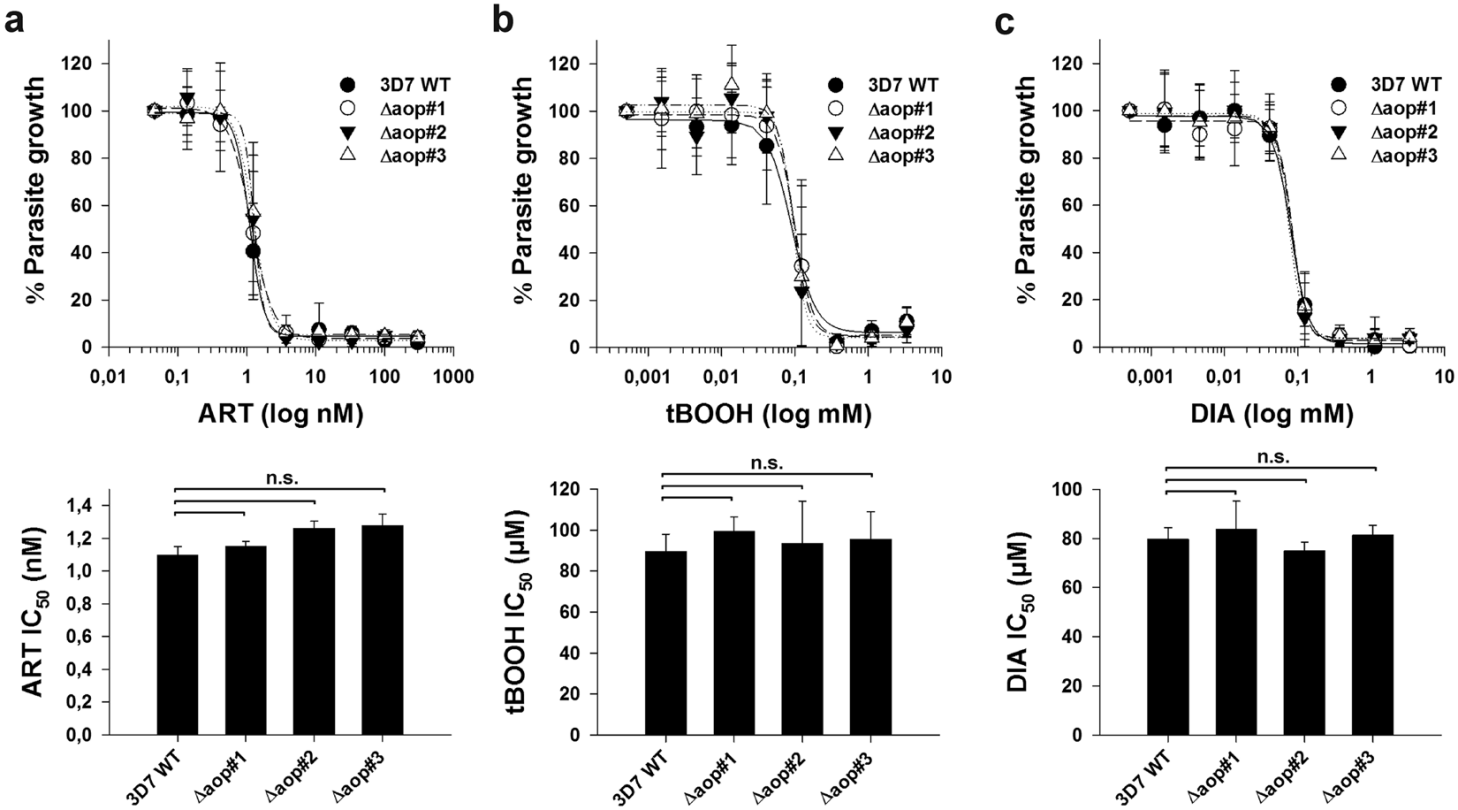
IC_50_ values for artemisinin and oxidants of Δ*pfaop* knockout parasites. (**a**) Artemisinin dose-response curves (upper panel) and IC_50_ values (lower panel) of blood stage cultures of strains Δaop#1-3 from Fig. 4. (b,c) tBOOH and diamide (DIA) dose-response curves (upper panel) and IC_50_ values (lower panel). All data are the mean ± standard deviation of at least three independent triplicate measurements. Wild type strain 3D7 served as a control. None of the differences between the IC_50_ values was found to be significant (p > 0.05) based on statistical analyses in SigmaPlot 12.5 using the one way ANOVA method.

To test whether the complete loss of *Pf*AOP affects the artemisinin susceptibility of *P. falciparum*, we cloned the plasmid pL7-*PFAOP* and deleted the encoding gene in strain 3D7 by double crossover using the CRISPR/Cas9 system^53^ (Fig. 4a). The genetic manipulation was monitored for three different clones following positive selection and limited dilution. All three clones had lost the endogenous gene and had integrated the selection marker after successful 5′- and 3′-crossovers as revealed by analytical PCR (Fig. 4b). Furthermore, the loss of *Pf*AOP was confirmed by western blotting using a specific antibody^41^ (Fig. 4c). Giemsa-stained blood smears of the three different knockout strains were subsequently analysed by light microscopy but revealed no suspicious morphologies. The growth rates were also identical to the wild type strain (Fig. 5). Next, we determined the IC_50_ values of the three knockout strains for artemisinin as well as the oxidants tBOOH and diamide as described previously^12, 54^. Loss of *Pf*AOP affected none of the IC_50_ values as compared to the wild type strain (Fig. 6). Please note that two different batches of artemisinin were used for the two independent sets of experiments in Figs 3 and 6, which presumably explains the systematic deviation of the IC_50_ value for artemisinin. The IC_50_ values for tBOOH in Fig. 6b (between 90 ± 8 µM and 99 ± 7 µM) and diamide in Fig. 6c (between 75 ± 4 µM and 84 ± 11 µM) were in accordance with previous measurements^54^.

**Figure 7:**
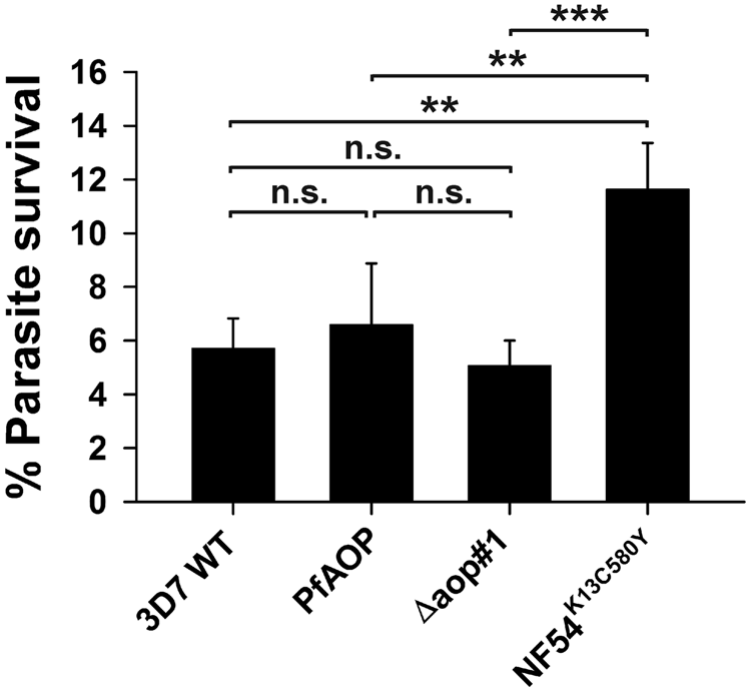
Ring-stage survival assays. Highly synchronous ring stage parasites were treated with or without 700 nM artemisinin for 6 h, washed and further incubated for 66 h. The parasitemia was subsequently determined from Giesma-stained blood smears. The parasite survival percentage was calculated for wild type strain 3D7 (3D7 WT), a strain overexpressing GFP-tagged full length PfAOP from Fig. 3 (PfAOP), a knockout strain from Fig. 4 (Δaop#1) and a positive control that carries the mutation for K13^C580Y^ (NF54^K13C580Y^). All data are the mean ± standard deviation of four independent experiments. Statistical analyses were performed in SigmaPlot 12.5 using the one way ANOVA method. Results are summarized on top of the bar chart (n.s., not significant; **p < 0.01; ***p < 0.001).

Recent studies revealed that ring-stage survival assays can be advantageous to address a potential artemisinin resistance, for example, because of short artemisinin half-lives and drug-induced parasite dormancy^55–58^. We therefore analyzed the ring-stage survival percentage of our knockout and overexpressing strains after treatment with 700 nM artemisinin. Wild type strain 3D7 and a mutant NF54 strain that encodes the resistance factor K13^C580Y^ were analyzed in parallel as negative and positive controls^53^, respectively (Fig. 7). Neither the deletion nor the overexpression of *PFAOP* had an effect on the ring-stage survival percentage, whereas the survival percentage of the positive control around 12% was twofold increased as compared to strain 3D7. (The difference between the controls was, in our hands, less pronounced as previously reported^53, 56–58^, most likely because we used 700 nM artemisinin instead of dihydroartemisininin in accordance with our QTL analysis, which was also performed with artemisinin). Taken together, the assays in Fig. 7 indicate that *Pf*AOP has no effect on the survival percentage of *P. falciparum* ring stage parasites under artemisinin pressure.

In summary, *Pf*AOP is not essential in asexual blood stage parasites and is dispensable for the removal of external oxidants such as tBOOH and diamide. Furthermore, the loss of *Pf*AOP taken alone does not affect the artemisinin susceptibility of *P. falciparum*. Thus, the correlation between the artemisinin susceptibility and chromosome 7 is either based on another gene within the identified locus or might require the interplay of several genetic factors.

## Discussion

Immunofluorescence microscopy and transcriptome analyses revealed that dual localized *Pf*AOP is constitutively expressed in blood stage parasites^33, 41^. What could be the function of *Pf*AOP in the apicoplast? Specific iron-sulfur clusters are, at least in prokaryotes, the major targets of oxidative challenges by superoxide anions and H_2_O_2_^59^. The apicoplast is essential for parasite survival because of the biosynthesis of the isoprenoid precursor isopentenyl pyrophosphate^60, 61^. Two enzymes of this biosynthetic pathway – the (*E*)-4-hydroxy-3-methylbut-2-enyl diphosphate synthase and reductase – bind iron-sulfur clusters^62, 63^. Interestingly, two of the five artemisinin susceptibility factors that were identified from Southeast Asian blood samples in the study by Miotto *et al*. localise to the apicoplast^10^. One of the apicoplast artemisinin susceptibility factors is ferredoxin, which also carries an iron-sulfur cluster^64, 65^. The iron-sulfur clusters of ferredoxin and the (*E*)-4-hydroxy-3-methylbut-2-enyl diphosphate synthase and reductase (as well as other apicoplast proteins) are probably synthesized by an essential machinery within the stroma of the organelle^50^. Considering the presence and relevance of redox-sensitive iron-sulfur cluster-containing apicoplast enzymes, it is quite surprising that the loss of the hydroperoxidase *Pf*AOP has no effect on parasite survival. One possible explanation could be the potential absence of major sources of superoxide anions or hydroperoxides in the apicoplast. However, the presence of an apicoplast superoxide dismutase^66^ seems to contradict this null hypothesis. Another explanation for the absent phenotype of our Δ*pfaop* knockout strains is that the cytosol and apicoplast harbor additional hydroperoxidases with redundant functions. Likely candidates in the cytosol are the dithiol Prx-isoform TPx-1, the Prx6-isoform 1-Cys-Prx and imported human Prx-2^37, 39, 40, 67^. The only alternative hydroperoxidase in the apicoplast appears to be the glutathione peroxidase-like thioredoxin peroxidase TPx_Gl_^68, 69^. At this point it is interesting to note that the expression of *Plasmodium* peroxidases might be interdependent and differ significantly depending on the growth conditions as reported for *P. berghei* ookinetes^70^. A third explanation for the absent knockout phenotype is that *Pf*AOP exerts another non-essential function, e.g., as a redox sensor that interacts with transducer molecules^29^. However, attempts to trap mixed disulfide bonds between *Pf*AOP and potential interaction partners so far only revealed false positive candidates (data not shown). In summary, the physiological function of dual localized *Pf*AOP remains unknown and the encoding gene is fully dispensable for the growth of asexual blood stage parasites in cell culture.

Neither traditional growth inhibition assays nor ring-stage survival assays revealed an altered artemisinin susceptibilty of *PFAOP* overexpressing and knockout strains. Since non-essential *PFAOP* on its own does not affect the artemisinin susceptibility, *PFAOP* might either contribute to a polygenic trait, or another gene within the identified locus on chromosome 7 might be the cause for the detected effects in Fig. 1. Which of the other genes from Table S1 might be relevant within the identified locus? Among the seven genes that were recently discovered as (potential) artemisinin susceptibility factors by chemogenomic profiling^11^, only one gene (PF3D7_0727100) is located on chromosome 7. PF3D7_0727100 encodes a conserved *Plasmodium* protein of unknown function, which was also identified in our QTL analysis (Table S1). It is therefore possible that PF3D7_0727100 is the crucial factor within the artemisinin susceptibility locus. Two other candidates from Table S1 – namely PF3D7_0727400, which encodes a putative proteasome subunit alpha type-5, and PF3D7_0729900, which encodes a putative dynein heavy chain – were also identified by mass spectrometry screens using modified artemisinin probes and click chemistry^16, 17^. PF3D7_0727400 might be of particular interest because of recent models that link artemisinin susceptibility to proteasome-dependent protein turnover^15, 18, 20, 71^. Future analyses are necessary to address the relevance of the candidates PF3D7_0727100, PF3D7_0727400 and PF3D7_0729900 for artemisinin susceptibility.

## Methods

### Materials

ART, DHA, ATS and DTBP were purchased from Sigma. ATM was from Sciphar. Recombinant *Pf*AOP with an N-terminal MRGSH_6_GS-tag replacing the first 59 amino acid residues of the full length protein as well as *P. falciparum* glutathione reductase (*Pf*GR) and *P. falciparum* glutaredoxin (*Pf*Grx) were produced in *Escherichia coli* and purified by affinity chromatography as described previously^45, 72^. Plasmid pUF1-Cas9 for the expression of Cas9 and plasmid pL6 for guide RNA expression and homologous recombination in *P. falciparum* ^53^ were kind gifts from J.J. Lopez-Rubio. Primers were from Metabion. WR99210 was a gift from Jacobus Pharmaceutical Company.

### Steady-state kinetics

Coupled enzymatic assays containing recombinant *Pf*AOP, *Pf*GR and *Pf*Grx were carried out at 25 °C using a thermostatted Jasco V-650 UV-visual spectrophotometer as described previously^45, 47^. All assays were performed in assay buffer containing 0.1 M Tris-HCl, 1 mM EDTA, pH 8.0. Stock solutions of 4 or 6 mM NADPH, 2 or 4 mM tBOOH, 40 mM GSH and 10 mM ATS were freshly prepared in assay buffer before each experiment. Stock solutions of 10 mM ART, ATM, DHA and DTBP were dissolved in DMSO. Whether *Pf*AOP converts endoperoxide substrates was tested in standard assays containing 150 µM NADPH, 1 U/mL *Pf*GR, 1 mM GSH and 5 µM *Pf*Grx. The consumption of NADPH was monitored at 340 nm (ε = 6.22 mM^−1^cm^−1^). A baseline was recorded for 60 sec before the addition of 100 µM ART, ATM, DHA, ATS or DTBP. All assays were subsequently started by adding 2.5 µM recombinant *Pf*AOP. Assays containing 100 µM tBOOH with or without the same volume of DMSO served as positive controls. Initial activities were corrected by subtracting the final slope (ΔAbs/min) of the baseline using the Spectra Analysis program (Spectra Manager version 2, Jasco).

To test whether endoperoxides are reversible inhibitors of *Pf*AOP, standard assays containing 50 µM tBOOH were supplemented with 0–100 µM ART, ATM, DHA, ATS or DTBP before starting the assay with 2.5 µM recombinant *Pf*AOP. The activity was determined and normalized to the negative controls without endoperoxide. A potential irreversible inhibition of *Pf*AOP was analysed in a time-course experiment. Samples containing 50 µM recombinant *Pf*AOP with or without 100 µM ART, ATM, DHA, ATS, DTBP or tBOOH were preincubated on ice for up to 10 min before transferring 50 µL of the mixture to a standard 1 mL assay with 100 µM tBOOH as a substrate. The residual activity was determined in the standard assay and normalized to the activity of negative controls that were preincubated in parallel without peroxide.

### Cloning of expression- and knockout constructs

The generation of pARL plasmids for the expression of full length, truncated and mutated fusion constructs between *Pf*AOP and GFP has been described previously^41^. Plasmid pL7-*PFAOP* was generated as follows: 1) A suitable guide sequence was identified in *PfAOP* with a confidence value of 0.010 using the protospacer software (http://www.protospacer.com). 2) The 3′-homology region of *PfAOP* was PCR-amplified using primers PfAOP/3′MCS/pL6/KO/s (5′-GATC*GAATTC*CAAACGATTTTACTTCAATAGATAC-3′) and PfAOP/3′MCS/pL6/KO/as (5′-GATC*CCATGG*CTGATTATTTTTTAAAAACTCTTTTAC-3′) and plasmid pQE30/*PFAOP* ^*C117S*^ as a template^45^. The PCR product was first subcloned according to the manufacturer’s protocol using the TopoTA cloning kit (Invitrogen) and subsequently cloned into pL6 using the restriction sites *Eco*RI and *Nco*I. 3) The 5′-homology region of *PfAOP* was PCR-amplified from genomic DNA of strain 3D7 using primers PfAOP/5′MCS/KO/s (5′-GATC*CCGCGG*CTGTTCTTTTTATTATATGAATGAAGAG-3′) and PfAOP/5′MCS/KO/as (5′-GATC*TCTAGA*GGATTCATAAACTTTTTGGGAAAACC-3′). The PCR product was first subcloned into the Topo cloning vector and subsequently cloned into pL6 using *Sac*II and the compatible restriction sites generated by *Xba*I (insert) and *Spe*I (pL6). 4) The guide sequence was cloned into pL6 yielding pL7-*PFAOP* as described previously^53^. Plasmid pL6 containing the 5′- and 3′-homology regions was digested with *Avr*II (2 h, 37 °C) and *Btg*ZI (2 h, 60 °C). The insert was generated by annealing oligonucleotide pL6/gRNA/PfAOP/s (5′-*TAAGTATATAATATT*ACAACATATCTGATACCGAT*GTTTTAGAGCTAGAA*-3′) and the complementary reverse oligonucleotide pL6/gRNA/PfAOP/as. (The 15 bp stretches that are homologous to pL6 and that flank the 20 bp guide sequence are highlighted in the sequence). The insert was mixed with digested pL6 and fused using the In-Fusion HD Cloning Kit (Clontech) according to the manufacturer’s protocol. Correct sequences of the intermediate and final constructs were confirmed by analytic restriction digests and commercial sequencing (GATC Biotech).

### Parasite culture, QTL analysis and genetic manipulation

*P. falciparum* strain 3D7 was cultured at 37 °C according to standard protocols^73^ using fresh human A^+^ erythrocytes at a hematocrit of 3% in RPMI 1640 medium that was supplemented with 0.45% (w/v) albumax II, 0.2 mM hypoxanthine and 2.7 μg/mL gentamicin. Progeny of the 7G8 × GB4 cross and the two parental clones were maintained at a hematocrit of 5% in RPMI 1640 medium supplemented with 10% inactivated human A^+^ serum instead of albumax II. Parasites were cultured at 80% humidity, 3 or 5% CO_2_, 5% O_2_ and 90% or 92% N_2_. Synchronization of ring stage parasites was carried out with 5% (w/v) sorbitol^74^. The F1 progeny from the GB4 × 7G8 cross were obtained from MR4^48^. The identity of all progeny was verified using eight polymorphic microsatellite markers^51^. QTL analysis was performed according to the method by Haley and Knott^75^ in line with previous independent studies^12, 51^. The genetic maps have been published^48^.

Transfections were performed as described^76^ using 100 μg of plasmid DNA per construct. Transfectants were usually detected in Giemsa-stained blood smears between two and four weeks post transfection. Parasites that were transfected with pARL plasmids were selected with 5 nM WR99210 and the expression of GFP-fusion constructs was confirmed by live cell imaging as described previously^41^. Parasites that were transfected with plasmid pUF1-Cas9 were selected with 100 nM atovaquone. After removal of atovaquone the strain was transfected with plasmid pL7-*PFAOP* and selected with 5 nM WR99210. Clonal parasite lines were obtained after limiting dilution and analysed by PCR and western blotting. Genomic DNA was isolated with phenol/chloroform as described previously^77^. Primers for analytical PCR reactions 1–3 in Fig. 4 were 3D7/P1/PfAOP/s (5′-ATATGATATATCTTATCGGTCCC-3′), probe/hDHFR/as (5′-CCTTTCTCCTCCTGGACATC-3′), probe/hDHFR/s (5′-CATGGTTCGCTAAACTGCATC-3′) and 3D7/P4/PfAOP/as (5′-TGGTATTACAAATAAGGGAAGAC-3′). Western blots against *Pf*AOP were performed using the purified peptide antibody αPfAOP-5 as described previously^41^ with slight modifications. Parasites were isolated without magnetic cell separation by treatment with 0.05% (w/v) saponin^78, 79^ in ice-cold buffer containing 1.84 mM KH_2_PO_4_, 10 mM Na_2_HPO_4_, 137 mM NaCl, 2.7 mM KCl, pH 7.4. Isolated parasites were resuspended in Laemmli buffer containing 30% (v/v) 2-mercaptoethanol, boiled for 10 min and analysed by SDS-PAGE and western blotting. Growth curves were determined for three asynchronous clonal knockout lines by counting approximately 1000 erythrocytes per Giemsa-stained blood smear.

### *In vitro* growth inhibition (IC_50_) assays

Cell proliferation assays in the presence of ART, ATM, tBOOH and diamide were performed using the SYBR Green method^49^ as described previously^12, 51, 54^. Synchronized ring stage parasites of overexpressing and knockout strains were incubated in 96-well plates with 1:3 serial drug dilutions. Drug concentrations ranged from 300 nM to 46 pM for ART and from 3.33 mM to 1 µM for tBOOH and diamide. Wells containing uninfected erythrocytes and infected erythrocytes without drug served as controls. Following incubation at 37 °C for 72 h, plates were frozen and stored at −80 °C. The plates were subsequently thawed at room temperature and the parasites were lysed with 2x lysis buffer containing 20 mM Tris-HCl, pH 7.5, 5 mM EDTA, 0.08% Triton X-100, 0.008% saponin and 0.12 µL/mL SYBR Green 1. The fluorescence was measured using a FLUOstar plate reader (BMG Labtech) with a gain set at 60 and an excitation and emission wavelength of 485 nm and 535 nm, respectively. Dose-response curves and IC_50_ values were computed using the four parameter Hill function in SigmaPlot 12.5. As a control, the *Pf*AOP content of overexpressing parasites was analyzed by standard western blotting and quantified using SuperSignal West Femto chemiluminiscent substrate (Thermo Scientific), a C-DiGit Blot Scanner and the software ImageStudioLite (LI-COR).

### Ring-stage survival assays

The *in vitro* ring-stage survival assay was adapted from Witkowski *et al*. ^56, 57^. Tightly sorbitol-synchronized early ring stage parasites (parasitemia 0.5–1%, hematocrit 2%, culture volume 2 mL) were treated in six well plates with 700 nM artemisinin for 6 h, washed five times with 5 mL RPMI, resuspended in complete medium and cultured for another 66 h. Blood smears were prepared, stained with Giemsa and approximately 10.000 erythrocytes were counted by light microscopy. Four independent measurements were conducted for each strain. Ring-stage survival percentages were calculated by comparing the number of viable parasites between drug-treated and mock-treated controls.

## Electronic supplementary material

Supplementary Information
