# Supplementary material for: Knockout of the peroxiredoxin 5 homologue PFAOP does not affect the artemisinin susceptibility of Plasmodium falciparum
Source: Sci Rep. 2017 Jun 30;7:4410. doi: 10.1038/s41598-017-04277-5 (PMC5493673; doi:10.1038/s41598-017-04277-5)
Supplement: Supplementary file 1 — Supplementary Information [file 41598_2017_4277_MOESM1_ESM.pdf]

Supplementary Information

Knockout of the peroxiredoxin 5 homologue *PFAOP* does not affect the artemisinin susceptibility of *Plasmodium falciparum*

Carine F. Djuika[1], Verena Staudacher[1], Cecilia P. Sanchez, Michael Lanzer, and Marcel Deponte*

Department of Parasitology, Ruprecht-Karls University, Im Neuenheimer Feld 324, D-69120 Heidelberg, Germany

[1]These authors contributed equally to this work.

*To whom correspondence should be addressed: Marcel Deponte, Department of Parasitology, Ruprecht-Karls University, Im Neuenheimer Feld 324, D-69120 Heidelberg, Germany; Tel: +49 6221 56 6518; Fax: +49 6221 56 4643; E-mail: marcel.deponte@gmx.de

Table S1. Annotated genes within the identified locus on chromosome 7 (strain 3D7).

Marker	Gene ID (new / old)	Annotation
C7MK70	PF3D7_0726100 / PF07_0107	Plasmodium exported protein, unknown function
	PF3D7_0726200 / MAL7P1.144	serine/threonine protein kinase, FIKK family
	PF3D7_0726300 / MAL7P1.145	mismatch repair protein pms1 homologue, putative
	PF3D7_0726400 / MAL7P1.146	conserved Plasmodium membrane protein, unknown function
	PF3D7_0726500 / MAL7P1.147	ubiquitin carboxyl-terminal hydrolase, putative
	PF3D7_0726600 / PF07_0108	conserved Plasmodium protein, unknown function
	PF3D7_0726700 / PF07_0109	conserved Plasmodium protein, unknown function
	PF3D7_0726800 / PF07_0109a	dolichyl-diphosphooligosaccharide--protein glycosyltransferase subunit DAD1, putative
	PF3D7_0726900 / PF07_0110	mitochondrial import inner membrane translocase subunit TIM50, putative
	PF3D7_0727000 / PF07_0111	vacuolar protein sorting-associated protein 53, putative
C7MK11	PF3D7_0727100 / MAL7P1.149	conserved Plasmodium protein, unknown function
	PF3D7_0727200 / MAL7P1.150	cysteine desulfurase, putative
	PF3D7_0727300 / MAL7P1.151	DNA (cytosine-5)-methyltransferase, putative
	PF3D7_0727400 / PF07_0112	proteasome subunit alpha type-5, putative
	PF3D7_0727500 / PF07_0113	conserved Plasmodium protein, unknown function
	PF3D7_0727600 / MAL7P1.152	conserved Plasmodium protein, unknown function
	PF3D7_0727700 / PF07_0114	conserved Plasmodium protein, unknown function
	PF3D7_0727800 / PF07_0115	cation transporting ATPase, putative
	PF3D7_0727900 / PF07_0116	conserved Plasmodium protein, unknown function
	PF3D7_0728000 / PF07_0117	eukaryotic translation initiation factor 2 alpha subunit, putative
	PF3D7_0728100 / PF07_0118	conserved Plasmodium membrane protein, unknown function
	PF3D7_0728200 / MAL7P1.153	actin-like protein, putative
	PF3D7_0728300 / PF07_0119	conserved Plasmodium protein, unknown function
	PF3D7_0728400 / MAL7P1.154	conserved Plasmodium protein, unknown function
	PF3D7_0728500 / MAL7P1.154a	conserved Plasmodium protein, unknown function
	PF3D7_0728600 / MAL7P1.155	zinc finger, C3HC4 type, putative
	PF3D7_0728700 / MAL7P1.156	alpha/beta-hydrolase, putative
	PF3D7_0728800 / MAL7P1.157	conserved Plasmodium protein, unknown function
	PF3D7_0728900 / MAL7P1.157a	RNA binding protein, putative
	PF3D7_0729000 / MAL7P1.158	signal recognition particle SRP9
	PF3D7_0729100 / PF07_0120	conserved Plasmodium protein, unknown function
	**PF3D7_0729200 / MAL7P1.159**	**1-cys peroxiredoxin**
	PF3D7_0729300 / PF07_0121	60S ribosomal export protein NMD3, putative
	PF3D7_0729400 / PF07_0122	BRIX protein, putative
	PF3D7_0729500 / PF07_0123	mRNA (N6-adenosine)-methyltransferase, putative
	PF3D7_0729600 / MAL7P1.160	conserved Plasmodium protein, unknown function
	PF3D7_0729700 / PF07_0124	conserved Plasmodium protein, unknown function

	PF3D7_0729800 / MAL7P1.161	dynein light chain, putative
	PF3D7_0729900 / MAL7P1.162	dynein heavy chain, putative
	PF3D7_0730000 / MAL7P1.163	conserved Plasmodium protein, unknown function
	PF3D7_0730100 / PF07_0125	tRNA pseudouridine synthase D, putative
	PF3D7_0730200 / MAL7P1.164	AP-4 complex subunit beta, putative
	PF3D7_0730300 / PF07_0126	transcription factor with AP2 domain(s), putative
	PF3D7_0730400 / PF07_0127	conserved Plasmodium protein, unknown function
	PF3D7_0730500 / MAL7P1.167	conserved Plasmodium protein, unknown function
	PF3D7_0730600 / MAL7_TRNA_VAL1	tRNA valine
	PF3D7_0730600 / MAL7_TRNA_THR2	tRNA threonine
BM99	PF3D7_0730800 / MAL7P1.170	Plasmodium exported protein, unknown function
	PF3D7_0730900 / MAL7P1.171	EMP1-trafficking protein
